# Functional polymorphisms of *NOS3* and *GUCY1A3* affect both nitric oxide formation and association with hypertensive disorders of pregnancy

**DOI:** 10.3389/fgene.2024.1293082

**Published:** 2024-02-26

**Authors:** Daniela A. Pereira, Marcelo R. Luizon, Ana C. Palei, José E. Tanus-Santos, Ricardo C. Cavalli, Valeria C. Sandrim

**Affiliations:** ^1^ Department of Genetics, Ecology and Evolution, Institute of Biological Sciences, Federal University of Minas Gerais, Belo Horizonte, Minas Gerais, Brazil; ^2^ Department of Biophysics and Pharmacology, Institute of Biosciences, Universidade Estadual Paulista (UNESP), Botucatu, Brazil; ^3^ Department of Surgery, University of Mississippi Medical Center, Jackson, MS, United States; ^4^ Department of Pharmacology, Ribeirao Preto Medical School, University of Sao Paulo, Ribeirão Preto, Brazil; ^5^ Department of Gynecology and Obstetrics, Ribeirao Preto Medical School, University of Sao Paulo, Ribeirão Preto, Brazil

**Keywords:** genetic polymorphisms, gestational hypertension, guanylate cyclase 1 soluble alpha 3, nitric oxide, nitric oxide synthase 3, preeclampsia, nitrite, pregnancy

## Abstract

Impaired nitric oxide (NO) formation may be associated with endothelial dysfunction and increased cardiovascular disease risk in preeclampsia (PE). Functional single-nucleotide polymorphisms (SNPs) of nitric oxide synthase 3 (*NOS3*) (rs3918226) and guanylate cyclase 1, soluble, alpha 3 (*GUCY1A3*) (rs7692387) increase susceptibility to the adverse consequences due to inadequate generation of NO by the endothelium. However, no previous study has examined whether these SNPs affect NO formation in healthy pregnancy and in gestational hypertension (GH) and PE. Here, we compared the alleles and genotypes of *NOS3* (rs3918226) and *GUCY1A3* (rs7692387) SNPs in normotensive pregnant women (NP, *n* = 153), in GH (*n* = 96) and PE (*n* = 163), and examined whether these SNPs affect plasma nitrite concentrations (a marker of NO formation) in these groups. We further examined whether the interaction among SNP genotypes is associated with GH and PE. Genotypes were determined using TaqMan allele discrimination assays, and plasma nitrite concentrations were determined by an ozone-based chemiluminescence assay. Multifactor dimensionality reduction was used to examine the interactions among SNP genotypes. Regarding *NOS3* rs3918226, the CT genotype (*p* = 0.046) and T allele (*p* = 0.020) were more frequent in NP than in GH, and GH patients carrying the CT+TT genotypes showed lower nitrite concentrations than NP carrying the CT+TT genotypes (*p* < 0.05). Regarding *GUCY1A3* rs7692387, the GA genotype (*p* = 0.013) and A allele (*p* = 0.016) were more frequent in PE than in NP, and NP women carrying the GG genotype showed higher nitrite concentrations than GH or PE patients carrying the GG genotype (*p* < 0.05). However, we found no significant interactions among genotypes for these functional SNPs to be associated with GH or PE. Our novel findings suggest that *NOS3* rs3918226 and *GUCY1A3* rs7692387 may affect NO formation and association with hypertensive disorders of pregnancy.

## 1 Introduction

Hypertensive disorders of pregnancy affect up to 10% of pregnancies (Hutcheon et al., 2011), which include gestational hypertension (GH) and preeclampsia (PE). PE is defined as new-onset hypertension after 20 weeks of pregnancy that occurs along with proteinuria or other indications of renal insufficiency, thrombocytopenia, liver dysfunction, pulmonary edema, and cerebral disturbances (ACOG, 2013). Therefore, PE is a complex multisystem disease and a major cause of maternal and perinatal mortality and morbidity (Chappell et al., 2021). Moreover, an increased risk of future cardiovascular disease has been observed in women with GH (Lo et al., 2020) and in women with a history of PE (Wu et al., 2017; Veiga et al., 2021), which can be explained by endothelial dysfunction during PE (Ahmed et al., 2014).

Widespread vascular endothelial dysfunction observed in PE is caused by the release of antiangiogenic factors into maternal circulation as a result of placental ischemia and hypoxia, including the soluble fms-like tyrosine kinase-1 (sFlt-1) (Maynard et al., 2003; Powe et al., 2011). It is to be noted that previous studies conducted by our research group found sFlt-1 to be inversely related to nitric oxide (NO) formation in PE, which is characterized by reduced bioavailability of NO (Sandrim et al., 2008b). In addition, we found that single-nucleotide polymorphisms (SNPs) of the endothelial NO synthase 3 (*NOS3*) gene may affect plasma and blood nitrite concentrations (a marker of endogenous NO formation) in healthy subjects (Metzger et al., 2013) and in patients with PE (Sandrim et al., 2010), and these SNPs of *NOS3* were found to be associated with susceptibility to GH or PE (Sandrim et al., 2008a; Muniz et al., 2012).

NO signaling pathway plays key roles in the regulation of vascular tone and platelet activation (Emdin et al., 2018). Notably, NO produced by the NOS3 enzyme is an effective vasodilator, with a key role in the control of blood pressure (Vanhoutte, 2003; Moncada and Higgs, 2006). NO activates soluble guanylyl cyclase, a heterodimeric protein with a subunit encoded by the guanylate cyclase 1, soluble, alpha 3 (*GUCY1A3*) gene (Forstermann et al., 1994; Mergia et al., 2006). Soluble guanylyl cyclase then produces cyclic guanosine monophosphate (cGMP), which activates downstream signaling molecules that lead to vasodilation, inhibition of platelet aggregation, and lowering of blood pressure (Denninger and Marletta, 1999). Therefore, NOS3 and GUCY1A3 are key mediators of NO signaling and its downstream effects (Emdin et al., 2018).

A remarkable study explored >335,000 participants in the UK Biobank genotyped using Affymetrix arrays, along with analysis from seven large-scale GWAS including nearly 1.5 million subjects, and found that variants that increase NO and NO-mediated cGMP synthesis are associated with decreased mean arterial pressure and reduced risk of coronary heart disease, peripheral arterial disease, and stroke (Emdin et al., 2018). Notably, common functional SNPs of *NOS3* (rs3918226) and *GUCY1A3* (rs7692387) were highlighted among these several array SNPs, which confirmed the adverse consequences of inadequate NO generation by the endothelium (Emdin et al., 2018; Loscalzo, 2018). However, no previous study has examined whether these functional SNPs of *NOS3* and *GUCY1A3* affect NO formation in healthy pregnancy and in GH or PE, including their association with GH or PE. Moreover, since *NOS3* and *GUCY1A3* are key genes that mediate NO signaling, they may interact to generate NO. However, no previous study has examined whether the interaction among their genotypes is associated with GH and PE.

In this study, we examined the association of these functional SNPs of *NOS3* (rs3918226) and *GUCY1A3* (rs7692387) with susceptibility to GH and PE and whether they affect NO formation in normotensive pregnant (NP) women and in patients with GH and PE. Moreover, we examined whether the interaction among genotypes for these functional SNPs is associated with GH and PE.

## 2 Methods

### 2.1 Study population

All volunteers were enrolled at the Department of Obstetrics and Gynecology, University Hospital at Ribeirao Preto Medical School of University of Sao Paulo (FMRP/USP). The Institutional Review Board at FMRP/USP approved this study. We included normotensive women with uncomplicated pregnancies (NP, *n* = 153) and patients with GH (*n* = 96) and PE (*n* = 163). GH was defined as pregnancy-induced hypertension (≥140 mmHg systolic or ≥90 mmHg diastolic blood pressure on two or more measurements at least 6 h apart) after 20 weeks of gestation and returning to normal by 12 weeks post-partum. PE was defined as GH along with significant proteinuria (≥0.3 g/L per 24 h) or with renal insufficiency, thrombocytopenia, liver dysfunction, pulmonary edema, and cerebral or visual symptoms (ACOG, 2013). The exclusion criteria of the study were women with pre-existing hypertension, with/without superimposed PE, and women with gestational diabetes. All individuals who were enrolled in this study are unrelated.

Written informed consent was provided by volunteers during clinic attendance. Maternal venous blood samples were collected into tubes containing EDTA and heparin, which were used for DNA extraction and to measure nitrite, respectively. Plasma samples were obtained after centrifugation at 1000 g for 10 min and stored at −70°C until assayed. Genomic DNA was extracted using a salting-out method from the cellular fraction of 1 mL of whole blood and stored at −20°C until analyzed.

### 2.2 Genotyping

Genotypes for *NOS3* rs3918226 and *GUCY1A3* rs7692387 were determined using TaqMan allelic discrimination assays (IDs C__30245515_10, and C__29125113_10, respectively; Applied Biosystems, Carlsbad, CA, USA). Real-time PCR was performed in a total volume of 12 μL (3 ng of template DNA, 1x TaqMan Genotyping Master Mix (Life Technologies Corporation, Grand Island, NY, USA) and 1x TaqMan allelic discrimination assay). Thermal cycling was performed in standard conditions, fluorescence was recorded by StepOnePlus Real-Time PCR equipment (Applied Biosystems, Carlsbad, CA, USA), and the results were obtained using the manufacturer’s software.

### 2.3 Measurement of nitrite concentrations

Nitrite concentrations were measured using an ozone-based chemiluminescence assay, as previously described (Sandrim et al., 2008b). Briefly, 200 μL of plasma aliquots analyzed in triplicate was injected into a solution of acidified triiodide, purging with nitrogen in-line with a gas-phase chemiluminescence NO analyzer (Sievers Model 280 NO Analyzer, General Electric Company, Boulder, CO, USA). Approximately 8 mL of triiodide solution (2.0 g of potassium iodide and 1.3 g of iodine dissolved in 40 mL of water with 140 mL of acetic acid) was placed in the purging vessel to which plasma samples were added. The triiodide solution reduced nitrite to NO gas, which was detected by the NO analyzer.

### 2.4 Statistical analysis

The characteristics of NP and GH and PE patients were compared using Student’s unpaired t-test, Mann–Whitney U test, or *x* ^2^ test as appropriate. The effects of different genotypes for *NOS3* and *GUCY1A3* SNPs on circulating nitrite concentrations among and within study groups were compared using one-way ANOVA (Kruskal–Wallis) with post-hoc tests (Dunn’s multiple comparison). Distribution of genotypes was assessed for deviation from Hardy–Weinberg equilibrium using Hardy–Weinberg exact tests for each locus in each population using the “web version of Genepop” available at https://genepop.curtin.edu.au/genepop_op1.html, which generates the estimation of exact *p*-values by the Markov chain method. We have used the suboptions “For each locus in the population” and “3. Probability test,” and the Markov chain parameters (default values): dememorization: 1000; batches: 100; iterations per batch: 1000 (Rousset, 2008) and differences in genotype and allele frequencies were assessed using *x* ^2^ tests. The difference in plasma nitrite concentrations among the study groups was obtained using one-way ANOVA followed by Bonferroni’s post-hoc test. The relationships between plasma nitrite concentrations and fasting glucose were analyzed using Spearman’s correlation (*r* and *p* values). A value of *p* < 0.05 was considered the level of statistical significance. Power calculation was performed using QUANTO version 1.2.4 (Gauderman, 2002). Given the sample size of the study, the power was 0.824 considering an alpha of 0.05 and the frequency of the minor allele of SNP *GUCY1A3* (rs7692387, G>A), which was found to be significantly associated with PE. The power was 0.721 considering an alpha of 0.05 and the frequency of the minor allele of SNP NOS3 (rs3918226, C>T), which was found to be significantly more frequent in NP than in GH.

Multifactor dimensionality reduction (MDR) characterizes the interactions of genotypes for their ability to be classified into different groups through cross-validation (CV) steps and permutation testing (Ritchie and Motsinger, 2005; Pander et al., 2010). The interaction model with the highest testing score and CV consistency (CVC) was considered the best interaction model, and its statistical significance was determined using permutation testing (Ritchie and Motsinger, 2005; Luizon et al., 2014; Luizon et al., 2017).

## 3 Results

The characteristics of subjects enrolled in this study are shown in Table 1. NP and GH and PE patients showed similar ethnicity, current smokers, heart rate, hemoglobin, hematocrit, creatinine, primiparity, and gestational age at sampling (all *p* > 0.05). As expected, PE and GH patients had higher systolic and diastolic blood pressure than NP patients (both *p* < 0.05), despite that most of these patients were receiving antihypertensive drugs. Patients with PE and GH were older than those with NP (*p* < 0.05). Increased body mass index and fasting glucose were found in both PE and GH compared with NP patients (both *p* < 0.05). Gestational age at delivery and newborn weights were lower in PE patients than in GH patients and NP (all *p* < 0.05). Significant proteinuria was found in PE.

**TABLE 1:** Clinical and demographic characteristics of the study subjects.

	Normotensive pregnant	Gestational hypertension	*p* ^*a*^	Preeclampsia	*p* ^*b*^	*p* ^*c*^
Parameter	NP (*n* = 153)	GH (*n* = 96)		PE (*n* = 163)		
Age (years)	24.5 ± 0.5	26.5 ± 0.7	**0.018**	26.2 ± 0.5	**0.020**	0.880
Ethnicity (% White)	59.45	73.96	0.305	67.49	0.527	0.690
Current smokers (%)	11.7	10.4	0.840	9.8	0.719	1.000
BMI (Kg m^−2^)	28.0 ± 0.3	33.8 ± 0.7	**0.000**	32.2 ± 0.51	**0.000**	0.067
SBP (mmHg)	111.0 ± 0.9	135.4 ± 1.76	**0.000**	142.4 ± 1.8	**0.000**	**0.001**
DBP (mmHg)	71.9 ± 0.8	85.0 ± 1.3	**0.000**	88.8 ± 1.0	**0.000**	**0.021**
HR (beats per min)	82.2 ± 0.8	82.1 ± 0.9	0.960	82.2 ± 0.7	0.727	0.873
Fasting glucose (mg/dL)	75.9 ± 1.3	83.4 ± 2.2	**0.009**	86.8 ± 2.6	**0.003**	0.747
Hb (mg/dL)	11.6 ± 0.19	12.0 ± 0.1	0.302	11.9 ± 0.1	0.340	0.845
Hct (%)	35.2 ± 0.52	36.0 ± 0.4	0.427	35.8 ± 0.4	0.453	0.808
Creatinine (mg/dL)	0.7 ± 0.0	0.63 ± 0.0	0.081	0.7 ± 0.0	0.688	**0.006**
24 h Pr (mg per 24 h)	ND	146.7 ± 13.5	**-**	1,474 ± 191.3	**-**	**0.000**
Primiparity (%)	49.2	41.0	0.477	42.5	0.529	0.907
GAD (weeks)	39.7 ± 0.1	38.9 ± 0.2	**0.001**	35.9 ± 0.3	**0.000**	**0.000**
Newborn weight (g)	3,291 ± 49.0	3,087 ± 56.1	**0.002**	2,549 ± 100.7	**0.000**	**0.000**
GAS (weeks)	36.2 ± 0.4	34.6 ± 0.5	**0.008**	35.1 ± 0.4	0.107	0.264

Abbreviations: BMI, body mass index; DBP, diastolic blood pressure; GAD, gestational age at delivery; GAS, gestational age at sampling; Hb, hemoglobin concentration; Hct, hematocrit; HR, heart rate; SBP, systolic blood pressure; ND: not determined (however, negative dipstick test). 24-h Pr, 24-h proteinuria.

Values are the mean ± SEM.

*p* ^a^ < 0.05: Gestational hypertension versus normotensive pregnant women.

*p* ^b^ < 0.05: Preeclampsia versus normotensive pregnant women.

*p* ^*c*^ < 0.05: Preeclampsia versus gestational hypertension.

Significant *p* values are in bold.

**FIGURE 1:**
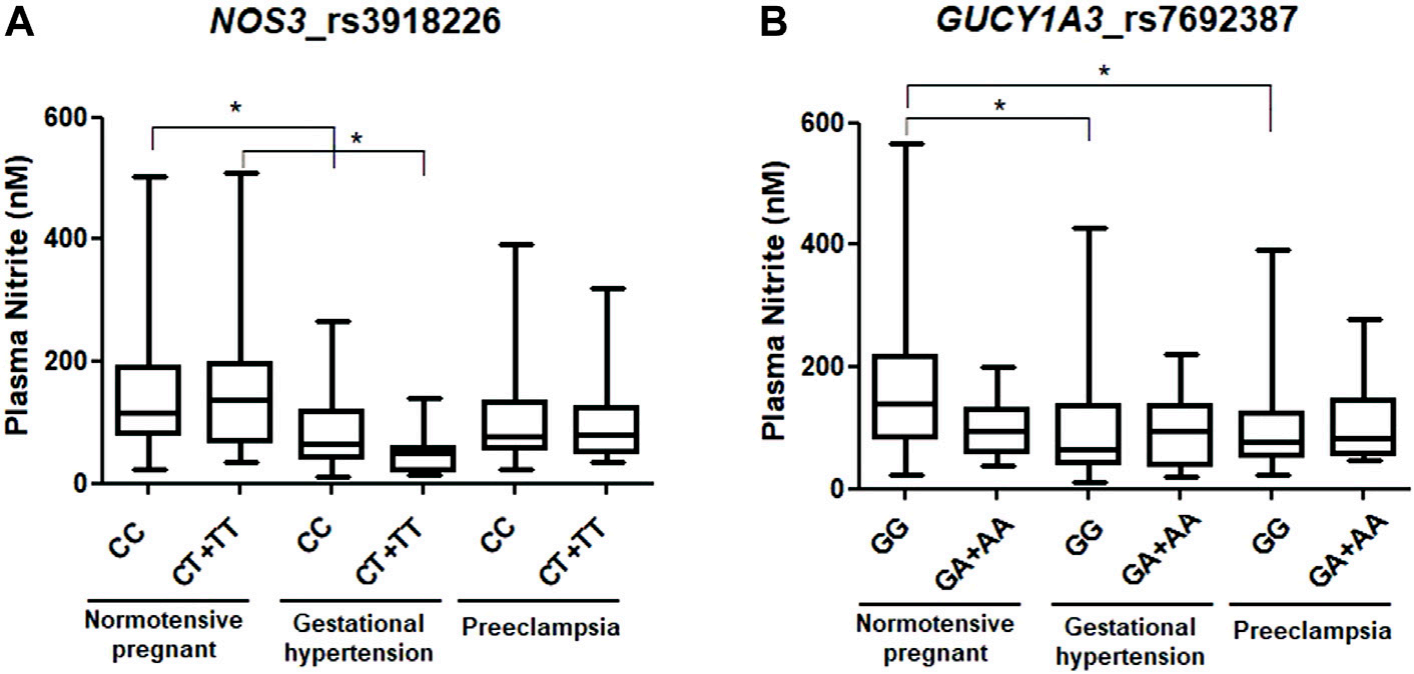
Effects of genotypes of **(A)** *NOS3* rs3918226 C>T and **(B)** *GUCY1A3* rs7692387 G>A SNPs on circulating nitrite concentrations in normotensive pregnant women and in patients with gestational hypertension and preeclampsia. *p < 0.05.

Genotype and allele distributions for NP, GH, and PE are shown in Table 2. The distribution of genotypes for each SNP in each of the study groups showed no deviation from the Hardy–Weinberg equilibrium (all *p* > 0.05). Regarding the *NOS3* rs3918226 SNP, the CT genotype and the T allele were more frequent in NP than in GH patients (both *p* < 0.05, and odds ratio (OR) = 0.435 and 0.407, respectively; Table 2). Considering the dominant model, the CT+TT genotypes were more frequent in NP than in GH patients (*p* = 0.032, OR = 0.407; Table 2). Regarding the *GUCY1A3* rs7692387 SNP, the GA genotype and the A allele were more frequent in PE patients than in NP patients (*p* < 0.05, OR = 1.991 and OR = 1.742, respectively; Table 2). Considering the dominant model, the GA+AA genotypes were more frequent in PE patients than in NP patients (*p* = 0.009, OR = 1.955; Table 2). We examined the effects of *NOS3 and GUCY1A3* genotypes on circulating nitrite concentrations. Due to no availability of plasma samples, circulating nitrite concentrations were measured only in a small number of subjects, NP (*n* = 95), GH (*n* = 41), and PE (*n* = 69). Plasma nitrite concentrations in the three study groups are shown in Supplementary Figure S1, which were found to be lower in both groups of hypertensive disorders of pregnancy (GH and PE) than in NP (both *p* < 0.05), as previously reported (Sandrim et al., 2008b). Regarding *NOS3* rs3918226, GH patients carrying both the CC and CT+TT genotypes showed lower nitrite concentrations than NP patients carrying the CC and CT+TT genotypes (*p* < 0.05, Figure 1A). Regarding *GUCY1A3* rs7692387, NP patients carrying the GG genotype showed higher nitrite concentrations than GH and PE patients carrying the GG genotype (*p* < 0.05, Figure 1B). However, we found no significant effects of different genotypes for both SNPs on nitrite concentrations within each of the study groups (*p* > 0.05).

**TABLE 2:** Genotype and allele frequencies for *NOS3* and *GUCY1A3* polymorphisms in normotensive pregnant women and in patients with gestational hypertension and preeclampsia.

*Gene* (SNP)	Normotensive pregnant (NP, *n* = 153)	Gestational hypertension (GH, *n* = 96)	OR (95% CI)	*p* ^*a*^	Preeclampsia (PE, *n* = 163)	OR (95% CI)	*p* ^*b*^
Genotype and allele							
*NOS3* (rs3918226, C>T)							
Codominant							
CC	122 (80%)	87 (91%)	1.000 (reference)	-	140 (86%)	1.000 (reference)	-
CT	29 (19%)	9 (9%)	0.435 (0.196–0.966)	0.046*	23 (14%)	0.691 (0.380–1.258)	0.229
TT	2 (1%)	0 (%)	0.280 (0.013–5.909)	0.513	0 (0%)	0.174 (0.008–3.670)	0.220
Dominant							
CC	122 (80%)	87 (91%)	1.000 (reference)	-	140 (86%)	1.000 (reference)	-
CT+TT	31 (20%)	9 (9%)	0.407 (0.185–0.899)	0.032*	23 (14%)	0.647 (0.358–1.168)	0.178
Alleles							
C	273 (89%)	183 (95%)	1.000 (reference)	-	303 (93%)	1.000 (reference)	-
T	33 (11%)	9 (5%)	0.407 (0.190–0.871)	0.020*	23 (7%)	0.628 (0.360–1.096)	0.123
*GUCY1A3* (rs7692387, G>A)							
Codominant							
GG	120 (78%)	68 (71%)	1.000 (reference)	-	106 (65%)	1.000 (reference)	-
GA	29 (19%)	27 (28%)	1.643 (0.899–3.00)	0.120	51 (31%)	1.991 (1.177–3.367)	0.013*
AA	4 (3%)	1 (1%)	0.441 (0.048–4.030)	0.657	6 (4%)	1.698 (0.466–6.182)	0.524
Dominant							
GG	120 (78%)	68 (71%)	1.000 (reference)	-	106 (65%)	1.000 (reference)	-
GA+AA	33 (22%)	28 (29%)	1.497 (0.834–2.688)	0.178	57 (35%)	1.955 (1.183–3.231)	0.009*
Alleles							
G	269 (88%)	163 (85%)	1.000 (reference)	-	263 (81%)	1.000 (reference)	-
A	37 (12%)	28 (15%)	1.249 (0.736–2.118)	0.415	63 (19%)	1.742 (1.121–2.705)	0.016*

Abbreviations: CI, confidence interval; *NOS3*, nitric oxide synthase 3; *GUCY1A3*, guanylate cyclase 1 soluble alpha 3; OR, odds ratio.

*p* ^a^ < 0.05: Gestational hypertension *versus* normotensive pregnant women.

*p* ^b^ < 0.05: Preeclampsia *versus* normotensive pregnant women.

Significant *p* values* are in bold.

We further examined the relationships between plasma nitrite concentrations and fasting glucose in the study groups (Supplementary Figure S2); however, we found no significant correlations.

Since *NOS3* and *GUCY1A3* are key genes that mediate NO signaling, it is possible that they may interact to generate NO. Therefore, we further examined whether the possible interaction among their genotypes was associated with susceptibility to GH and PE. However, we found no model of interaction among genotypes of *NOS3* (rs3918226) and *GUCY1A3* (rs7692387) SNPs to be significantly associated with GH or with PE (*p* > 0.05, Table 3).

**TABLE 3:** MDR interaction models among *NOS3* and *GUCY1A3* polymorphisms in normotensive pregnant (NP) compared with both gestational hypertension (GH) and with preeclampsia (PE).

Interaction model	Training score	Testing score	CVC	*p*
NP (*n* = 153) vs. GH (*n* = 88)				
*NOS3* rs3918226 C>T	0.534	0.550	9/10	0.145–0.146
*NOS3* rs3918226 C>T, *GUCY1A3* rs7692387 G>A	0.599	0.556	10/10	0.110
NP (*n* = 153) vs. PE (*n* = 163)				
*GUCY1A3* rs7692387 G>A	0.566	0.559	10/10	0.056
*NOS3* rs3918226 C>T, *GUCY1A3* rs7692387 G>A	0.558	0.504	10/10	0.638–0.639

Abbreviations: CVC, cross-validation consistency; NOS3, nitric oxide synthase 3; GUCY1A3, guanylate cyclase 1, soluble, alpha 3; MDR, multifactor dimensionality reduction; PE, preeclampsia; GH, gestational hypertension; NP, normotensive pregnant.

*p < 0.05.

## 4 Discussion

The main novel findings reported here are that the CT genotype and T allele for *NOS3* rs3918226 were more frequent in NP than in GH patients, and GH patients carrying the CT+TT genotypes showed lower nitrite concentrations than NP patients carrying the CT+TT genotypes. Regarding *GUCY1A3* rs7692387, the GA genotype and A allele were more frequent in PE patients than in NP, and NP patients carrying the GG genotype showed higher nitrite concentrations than GH or PE patients carrying the GG genotype. However, no significant model of interaction among genotypes of these functional *NOS3* and *GUCY1A3* SNPs was associated with GH or with PE.

Common variants in the *NOS3* promoter (rs3918226) and *GUCY1A3* intron (rs7692387) were associated with increased *NOS3* and *GUCY1A3* expression and with reduced mean arterial pressure (Emdin et al., 2018). The effects of *NOS3* rs3918226 on NO formation were previously examined in healthy subjects (Luizon et al., 2012), and no significant differences were found when comparing the nitrite concentrations in the CC genotype with the CT+TT genotypes (Luizon et al., 2012). However, the *NOS3* rs3918226 was previously associated with NO production in children and adolescents (de Miranda et al., 2015). rs3918226 is located next to a potential transcription factor-binding site for the Ets family domain, and it may affect transcription factor-binding affinity (Salvi et al., 2012). Indeed, it was previously shown to affect *NOS3* expression using luciferase assays in HeLa and HEK293T cells transfected with the *NOS3* promoter carrying either the T or the C allele (Salvi et al., 2013). The T allele was associated with reduced *NOS3* transcriptional activity compared with the C allele of the rs3918226 located at the *NOS3* promoter (Salvi et al., 2013). Moreover, the TT homozygosity was associated with lesser transcription and higher hypertension risk (Salvi et al., 2013). In the Genome Tissue Expression (GTEx) project database (Consortium, 2015), the C allele was associated with increased *NOS3* expression in the lung tissue. In the present study, we find that GH patients carrying the CT+TT genotypes showed lower nitrite concentrations than NP patients carrying the CT+TT genotypes. However, we found no difference within each of the study groups.

To our knowledge, no previous study has examined the association of *GUCY1A3* polymorphisms with PE. We showed for the first time that the GA genotype and A allele for the *GUCY1A3* rs7692387 were more frequent in PE patients than in NP. Consistent with this finding, the A allele of *GUCY1A3* rs7692387 was associated with hypertensive disorders of pregnancy (OR = 0.90 [95% CI, 0.84–0.97]; *p* = 0.004) (Honigberg et al., 2020). However, this study used phenotypic data from the UK Biobank, but it has not defined the pregnant women who had PE (Honigberg et al., 2020). Nevertheless, no previous study has examined the effects of *GUCY1A3* polymorphisms on NO formation in hypertensive disorders of pregnancy. The minor allele A of the rs7692387 polymorphism was associated with increased *GUCY1A3* expression in the GTEx portal (Consortium, 2015). Accordingly, the G allele of *GUCY1A3* rs7692387 has been characterized to reduce the expression of *GUCY1A3* via disruption of a ZEB1 transcription factor site (Kessler et al., 2017). However, we found no significant effects for the different *GUCY1A3* rs7692387 genotypes on circulating nitrite concentrations within each of the study groups. Despite that, NP patients carrying the GG genotype showed higher nitrite levels than those of GH and PE carrying the same genotype. The protective effect of variation on *GUCY1A3* rs7692387 against hypertensive disorders of pregnancy and the underlying pathways relative to blood pressure lowering requires further studies.

Since plasma nitrite levels were previously associated with body mass index (Ghasemi et al., 2008), fasting blood glucose, and systolic blood pressure (Li et al., 2004), we examined the potential relationships between plasma nitrite concentrations and fasting glucose in the three study groups. However, we found no significant correlations between plasma nitrite concentrations and fasting glucose in our study, which could be due to the small number of subjects with measurements for nitrite concentrations for NP and GH and PE. Further studies are needed to explore the correlations of plasma nitrite concentrations with fasting glucose in hypertensive disorders of pregnancy.

These studies show that SNPs of *NOS3* and *GUCY1A3* may individually be relevant to the susceptibility of PE, with a focus on the role of NO bioavailability in the physiopathology of PE. However, epistasis may also be relevant, and gene–gene interactions have been used before to detect susceptibility to complex diseases and response to drugs, including PE, (Gui et al., 2011; Williams and Broughton Pipkin, 2011; Singhal et al., 2023) and response to antihypertensive therapy (Luizon et al., 2018). Given that the final products of *NOS3* and *GUCY1A3* genes interact together in the same biological pathway (Wobst et al., 2018), the NO produced by NOS3 activates soluble guanylyl cyclase, which has a subunit encoded by GUCY1A3 (Forstermann et al., 1994; Mergia et al., 2006). Thus, it is of relevance to explore the interaction among its polymorphisms and how it could affect the susceptibility to hypertensive disorders of pregnancy. In this context, we examined whether the interactions among genotypes for the functional SNPs of *NOS3* (rs3918226) and *GUCY1A3* (rs7692387) were associated with GH and with PE. Although we found no significant model of interaction, further studies should be performed in order to examine the interactions among other polymorphisms in other candidate genes that are relevant to the physiopathology of PE.

The present study has limitations. The circulating nitrite concentrations were measured only in a small number of subjects enrolled due to no availability of plasma samples and technical reasons. Nevertheless, we were able to found significant effects of different genotypes for *NOS3* rs3918226 and *GUCY1A3* rs7692387 on nitrite concentrations in hypertensive disorders of pregnancy. Importantly, our findings must be replicated on different populations by further studies, which should consider an increase in the sample size. It is to be noted that allele frequencies for both SNPs analyzed (*NOS3*, rs3918226 and *GUCY1A3*, rs7692387) do not show important differences among Europeans, Africans, and Asians, according to the dbSNP database (Sherry et al., 2001). Finally, an increase in the number of the studied SNPs, at least in the *NOS3* gene, could also be worthy for examining the potential association of haplotypes with nitrite concentrations.

In conclusion, we found that the CT genotype and the T allele for *NOS3* rs3918226 were more frequent in NP than in patients with GH, while the GA genotype and A allele for *GUCY1A3* rs7692387 were more frequent in patients with PE than NP. Moreover, patients with GH carrying the CT+TT genotypes for *NOS3* rs3918226 showed lower nitrite concentrations than those with NP carrying the CT+TT genotypes, and NP patients carrying the GG genotype for *GUCY1A3* rs7692387 showed higher nitrite concentrations than patients with GH or with PE carrying the same GG genotype. However, we found no significant model of interaction among genotypes for these functional SNPs to be associated with GH or with PE. Our novel findings suggest that functional SNPs of *NOS3* (rs3918226) and *GUCY1A3* (rs7692387) may affect NO formation and be associated with hypertensive disorders of pregnancy.

## Data Availability

The original contributions presented in the study are included in the article/Supplementary Material; further inquiries can be directed to the corresponding authors.
