# Supplementary material for: Functional polymorphisms of NOS3 and GUCY1A3 affect both nitric oxide formation and association with hypertensive disorders of pregnancy
Source: Front Genet. 2024 Feb 26;15:1293082. doi: 10.3389/fgene.2024.1293082 (PMC10925623; doi:10.3389/fgene.2024.1293082)
Supplement: Supplementary file 1 [file DataSheet1.docx]

**SUPPLEMENTARY FIGURE 1**

**
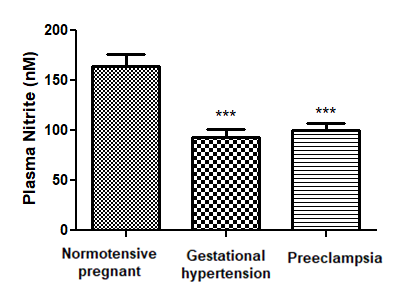
**

**Supplementary Figure 1.** Plasma nitrite levels in normotensive pregnant, gestational hypertension and preeclampsia patients. Bars indicates means ±SEMs. *** *P*<0.05 *vs* normotensive pregnant patients calculated using one-way ANOVA followed by Bonferroni’s post-hoc comparisons.

**SUPPLEMENTARY FIGURE 2**

**
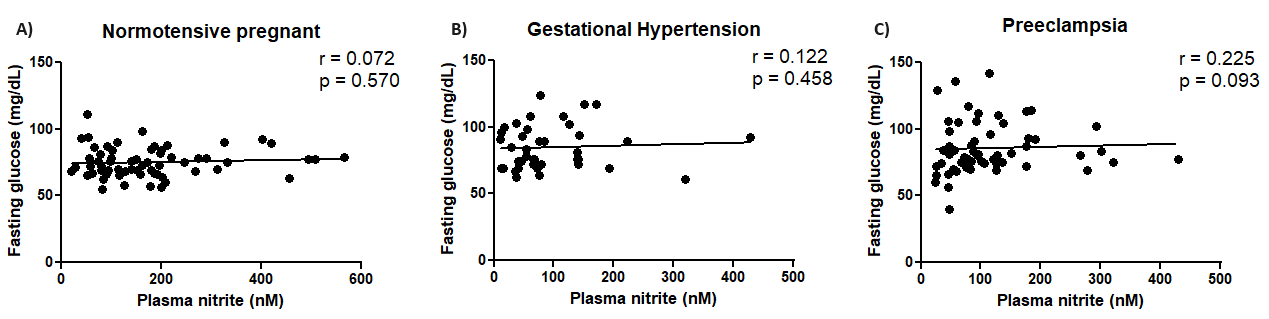
**

**Supplementary Figure 2.** Correlations between plasma nitrite levels (nM) and fasting glucose (mg/dL) in Normotense Pregnant **(A)**, Gestational Hypertension **(B)** and in Preeclampsia **(C)**. *P* value and Spearman’s correlation (r) are reported. Regression lines are plotted.

**Supplementary Material 1.** Input file of the Genepop analysis.

NOS3_GUCY1A3

rs3918226, rs7692387

Pop

PE, 0101 0101

PE, 0101 0101

PE, 0102 0102

PE, 0101 0102

PE, 0101 0101

PE, 0102 0101

PE, 0101 0102

PE, 0101 0101

PE, 0101 0101

PE, 0101 0101

PE, 0101 0101

PE, 0101 0101

PE, 0101 0101

PE, 0101 0102

PE, 0101 0101

PE, 0101 0102

PE, 0101 0102

PE, 0101 0101

PE, 0101 0101

PE, 0101 0101

PE, 0101 0101

PE, 0101 0102

PE, 0101 0101

PE, 0101 0102

PE, 0101 0102

PE, 0101 0101

PE, 0101 0101

PE, 0101 0101

PE, 0101 0101

PE, 0101 0101

PE, 0101 0101

PE, 0101 0102

PE, 0101 0102

PE, 0101 0102

PE, 0101 0101

PE, 0101 0101

PE, 0101 0102

PE, 0101 0101

PE, 0101 0101

PE, 0101 0102

PE, 0102 0101

PE, 0101 0101

PE, 0101 0101

PE, 0101 0101

PE, 0101 0102

PE, 0101 0102

PE, 0101 0101

PE, 0101 0101

PE, 0101 0101

PE, 0101 0102

PE, 0101 0101

PE, 0101 0101

PE, 0101 0101

PE, 0101 0101

PE, 0101 0101

PE, 0101 0101

PE, 0101 0101

PE, 0101 0102

PE, 0101 0101

PE, 0101 0102

PE, 0101 0101

PE, 0101 0101

PE, 0101 0102

PE, 0101 0102

PE, 0101 0101

PE, 0101 0101

PE, 0101 0101

PE, 0101 0101

PE, 0101 0101

PE, 0101 0102

PE, 0101 0101

PE, 0101 0101

PE, 0101 0101

PE, 0101 0102

PE, 0101 0102

PE, 0101 0102

PE, 0101 0101

PE, 0101 0101

PE, 0101 0202

PE, 0101 0102

PE, 0101 0102

PE, 0101 0102

PE, 0101 0202

PE, 0101 0202

PE, 0102 0101

PE, 0101 0102

PE, 0102 0102

PE, 0102 0101

PE, 0101 0101

PE, 0102 0101

PE, 0101 0101

PE, 0101 0101

PE, 0101 0102

PE, 0101 0101

PE, 0102 0102

PE, 0101 0102

PE, 0101 0101

PE, 0102 0101

PE, 0101 0101

PE, 0101 0101

PE, 0101 0101

PE, 0101 0101

PE, 0101 0101

PE, 0102 0101

PE, 0101 0101

PE, 0101 0101

PE, 0101 0102

PE, 0101 0202

PE, 0102 0101

PE, 0101 0101

PE, 0102 0101

PE, 0102 0101

PE, 0101 0101

PE, 0101 0101

PE, 0102 0101

PE, 0101 0101

PE, 0101 0202

PE, 0101 0102

PE, 0101 0102

PE, 0102 0102

PE, 0101 0101

PE, 0101 0101

PE, 0101 0101

PE, 0101 0102

PE, 0101 0101

PE, 0101 0101

PE, 0101 0101

PE, 0101 0101

PE, 0101 0101

PE, 0101 0101

PE, 0101 0101

PE, 0102 0102

PE, 0101 0101

PE, 0101 0101

PE, 0101 0102

PE, 0101 0102

PE, 0101 0202

PE, 0101 0102

PE, 0101 0101

PE, 0102 0102

PE, 0101 0101

PE, 0101 0101

PE, 0101 0102

PE, 0101 0101

PE, 0102 0101

PE, 0101 0101

PE, 0101 0102

PE, 0101 0102

PE, 0101 0101

PE, 0102 0102

PE, 0101 0101

PE, 0101 0102

PE, 0101 0101

PE, 0101 0102

PE, 0101 0101

PE, 0102 0101

PE, 0102 0101

PE, 0102 0101

PE, 0101 0101

PE, 0101 0102

PE, 0102 0102

PE, 0101 0101

PE, 0101 0101

Pop

GH, 0101 0101

GH, 0101 0101

GH, 0101 0101

GH, 0101 0101

GH, 0102 0101

GH, 0102 0102

GH, 0101 0101

GH, 0101 0101

GH, 0101 0101

GH, 0101 0102

GH, 0101 0101

GH, 0101 0101

GH, 0102 0101

GH, 0101 0101

GH, 0101 0101

GH, 0101 0101

GH, 0101 0101

GH, 0101 0101

GH, 0101 0101

GH, 0101 0101

GH, 0101 0101

GH, 0101 0101

GH, 0101 0101

GH, 0101 0102

GH, 0101 0101

GH, 0101 0101

GH, 0101 0101

GH, 0101 0101

GH, 0101 0101

GH, 0101 0102

GH, 0101 0101

GH, 0101 0102

GH, 0101 0101

GH, 0101 0101

GH, 0101 0202

GH, 0101 0102

GH, 0101 0101

GH, 0101 0102

GH, 0101 0101

GH, 0101 0101

GH, 0101 0101

GH, 0101 0101

GH, 0101 0101

GH, 0101 0102

GH, 0101 0101

GH, 0101 0102

GH, 0101 0101

GH, 0101 0101

GH, 0101 0101

GH, 0101 0101

GH, 0101 0101

GH, 0101 0101

GH, 0101 0101

GH, 0101 0101

GH, 0101 0102

GH, 0101 0102

GH, 0101 0101

GH, 0101 0101

GH, 0101 0101

GH, 0101 0101

GH, 0101 0101

GH, 0101 0102

GH, 0101 0101

GH, 0101 0101

GH, 0101 0102

GH, 0101 0101

GH, 0101 0102

GH, 0101 0101

GH, 0101 0102

GH, 0101 0101

GH, 0101 0101

GH, 0102 0102

GH, 0102 0101

GH, 0101 0102

GH, 0101 0102

GH, 0101 0101

GH, 0101 0102

GH, 0101 0102

GH, 0101 0102

GH, 0101 0101

GH, 0101 0101

GH, 0102 0102

GH, 0101 0102

GH, 0101 0102

GH, 0101 0102

GH, 0101 0101

GH, 0102 0101

GH, 0101 0101

GH, 0101 0101

Pop

Ctrl, 0000 0101

Ctrl, 0101 0101

Ctrl, 0000 0101

Ctrl, 0101 0101

Ctrl, 0101 0102

Ctrl, 0101 0102

Ctrl, 0101 0101

Ctrl, 0101 0101

Ctrl, 0101 0202

Ctrl, 0101 0101

Ctrl, 0101 0101

Ctrl, 0101 0102

Ctrl, 0101 0102

Ctrl, 0101 0101

Ctrl, 0101 0202

Ctrl, 0102 0101

Ctrl, 0101 0101

Ctrl, 0101 0101

Ctrl, 0101 0101

Ctrl, 0101 0101

Ctrl, 0102 0101

Ctrl, 0102 0102

Ctrl, 0101 0101

Ctrl, 0101 0101

Ctrl, 0101 0101

Ctrl, 0101 0101

Ctrl, 0101 0101

Ctrl, 0102 0101

Ctrl, 0101 0101

Ctrl, 0101 0102

Ctrl, 0101 0101

Ctrl, 0101 0101

Ctrl, 0102 0101

Ctrl, 0101 0101

Ctrl, 0102 0101

Ctrl, 0101 0101

Ctrl, 0101 0102

Ctrl, 0101 0101

Ctrl, 0101 0101

Ctrl, 0101 0101

Ctrl, 0101 0102

Ctrl, 0101 0101

Ctrl, 0101 0101

Ctrl, 0101 0101

Ctrl, 0101 0101

Ctrl, 0101 0101

Ctrl, 0101 0101

Ctrl, 0101 0102

Ctrl, 0102 0101

Ctrl, 0102 0102

Ctrl, 0101 0101

Ctrl, 0101 0102

Ctrl, 0101 0101

Ctrl, 0101 0101

Ctrl, 0102 0102

Ctrl, 0101 0101

Ctrl, 0101 0101

Ctrl, 0101 0202

Ctrl, 0101 0101

Ctrl, 0102 0101

Ctrl, 0101 0101

Ctrl, 0101 0101

Ctrl, 0101 0101

Ctrl, 0101 0101

Ctrl, 0101 0101

Ctrl, 0101 0101

Ctrl, 0101 0101

Ctrl, 0101 0101

Ctrl, 0101 0101

Ctrl, 0101 0101

Ctrl, 0101 0102

Ctrl, 0101 0101

Ctrl, 0102 0101

Ctrl, 0102 0101

Ctrl, 0102 0101

Ctrl, 0101 0101

Ctrl, 0202 0101

Ctrl, 0101 0101

Ctrl, 0101 0101

Ctrl, 0101 0101

Ctrl, 0102 0101

Ctrl, 0101 0101

Ctrl, 0102 0101

Ctrl, 0101 0101

Ctrl, 0101 0101

Ctrl, 0102 0101

Ctrl, 0102 0102

Ctrl, 0101 0102

Ctrl, 0101 0101

Ctrl, 0101 0101

Ctrl, 0102 0102

Ctrl, 0102 0101

Ctrl, 0101 0101

Ctrl, 0101 0101

Ctrl, 0101 0102

Ctrl, 0101 0101

Ctrl, 0101 0102

Ctrl, 0101 0101

Ctrl, 0101 0101

Ctrl, 0101 0102

Ctrl, 0101 0102

Ctrl, 0101 0101

Ctrl, 0101 0101

Ctrl, 0101 0101

Ctrl, 0101 0101

Ctrl, 0101 0101

Ctrl, 0101 0101

Ctrl, 0102 0101

Ctrl, 0102 0101

Ctrl, 0102 0101

Ctrl, 0101 0101

Ctrl, 0101 0101

Ctrl, 0102 0101

Ctrl, 0101 0102

Ctrl, 0101 0101

Ctrl, 0101 0101

Ctrl, 0101 0101

Ctrl, 0101 0101

Ctrl, 0102 0101

Ctrl, 0101 0101

Ctrl, 0101 0102

Ctrl, 0101 0101

Ctrl, 0101 0101

Ctrl, 0101 0101

Ctrl, 0101 0101

Ctrl, 0101 0101

Ctrl, 0101 0101

Ctrl, 0101 0102

Ctrl, 0101 0101

Ctrl, 0101 0101

Ctrl, 0101 0101

Ctrl, 0101 0202

Ctrl, 0102 0102

Ctrl, 0101 0102

Ctrl, 0101 0102

Ctrl, 0101 0102

Ctrl, 0101 0102

Ctrl, 0101 0101

Ctrl, 0101 0101

Ctrl, 0101 0101

Ctrl, 0102 0101

Ctrl, 0101 0101

Ctrl, 0101 0101

Ctrl, 0101 0101

Ctrl, 0101 0101

Ctrl, 0202 0101

Ctrl, 0101 0102

Ctrl, 0101 0101

Ctrl, 0102 0101

Ctrl, 0102 0101

Ctrl, 0101 0101

Ctrl, 0101 0101

Ctrl, 0102 0101

Ctrl, 0101 0101

Ctrl, 0101 0101

**Supplementary Material 2.** Output file of the Genepop analysis.

**Results from GENEPOP**

Mon Jan 29 22:33:37 AWST 2024

Genepop 4.7.5: Hardy-Weinberg test

File: 223337 (NOS3_GUCY1A3)

Number of populations detected: 3

Number of loci detected: 2

Estimation of exact P-Values by the Markov chain method.

---------------------------------------------

Markov chain parameters for all tests:

Dememorization: 1000

Batches: 100

Iterations per batch: 1000

Hardy Weinberg: Probability test

************************

==========================================

Results by locus

==========================================

Locus "rs3918226"

-----------------------------------------

Fis estimates

---------------

POP P-val S.E. W&C R&H Steps

----------- ------- ------- ------- ------- ------

PE 1.0000 0.0000 -0.0728 -0.0731 56545 switches

GH 1.0000 0.0000 -0.0353 -0.0355 11580 switches

Ctrl 0.6839 0.0019 0.0183 0.0183 68460 switches

All (Fisher's method):

Chi2: 0.7600

Df : 6

Prob : 0.993103

Locus "rs7692387"

-----------------------------------------

Fis estimates

---------------

POP P-val S.E. W&C R&H Steps

----------- ------- ------- ------- ------- ------

PE 1.0000 0.0000 -0.0004 -0.0004 80382 switches

GH 0.6839 0.0016 -0.0859 -0.0863 68036 switches

Ctrl 0.2363 0.0026 0.1132 0.1136 70130 switches

All (Fisher's method):

Chi2: 3.6451

Df : 6

Prob : 0.724570

==========================================

Results by population

==========================================

Pop : PE

-----------------------------------------

Fis estimates

---------------

locus P-val S.E. W&C R&H Steps

----------- ------- ------- ------- ------- ------

rs3918226 1.0000 0.0000 -0.0728 -0.0731 56545 switches

rs7692387 1.0000 0.0000 -0.0004 -0.0004 80382 switches

All (Fisher's method):

Chi2 : 0.0000

Df : 4

Prob : 1

Pop : GH

-----------------------------------------

Fis estimates

---------------

locus P-val S.E. W&C R&H Steps

----------- ------- ------- ------- ------- ------

rs3918226 1.0000 0.0000 -0.0353 -0.0355 11580 switches

rs7692387 0.6839 0.0016 -0.0859 -0.0863 68036 switches

All (Fisher's method):

Chi2 : 0.7600

Df : 4

Prob : 0.943728

Pop : Ctrl

-----------------------------------------

Fis estimates

---------------

locus P-val S.E. W&C R&H Steps

----------- ------- ------- ------- ------- ------

rs3918226 0.6839 0.0019 0.0183 0.0183 68460 switches

rs7692387 0.2363 0.0026 0.1132 0.1136 70130 switches

All (Fisher's method):

Chi2 : 3.6451

Df : 4

Prob : 0.456155

==========================================

All locus, all populations

==========================================

All (Fisher's method) :

Chi2 : 4.4051

Df : 12

Prob : 0.974968

Normal ending
